# In vitro virulence characteristics of rare serovars of *Salmonella enterica* isolated from sand lizards (*Lacerta agilis* L.)

**DOI:** 10.1007/s10482-018-1079-8

**Published:** 2018-05-19

**Authors:** Joanna Mokracka, Sylwia Krzymińska, Danił Ałtunin, Dariusz Wasyl, Ryszard Koczura, Krzysztof Dudek, Monika Dudek, Zofia Anna Chyleńska, Anna Ekner-Grzyb

**Affiliations:** 1 Department of Microbiology, Faculty of Biology, Adam Mickiewicz University in Poznań, Umultowska 89, 61-614 Poznan, Poland; 2 Department of Microbiology, National Veterinary Research Institute, Partyzantów 57, 24-100 Puławy, Poland; 3 Department of Zoology, Institute of Zoology, Poznań University of Life Sciences, Wojska Polskiego 71 C, 60-625 Poznan, Poland; 4 Laboratory of Neurobiology, Institute of Zoology, Poznań University of Life Sciences, Wojska Polskiego 71 C, 60-625 Poznan, Poland; 5 Department of Nature Education and Conservation, Faculty of Biology, Adam Mickiewicz University in Poznań, Umultowska 89, 61-614 Poznan, Poland; 6 Department of Plant Ecophysiology, Faculty of Biology, Adam Mickiewicz University in Poznań, Umultowska 89, 61-614 Poznan, Poland; 7 HiProMine S.A., ul. Poznańska 8, 62-023 Robakowo, Poland

**Keywords:** Pathogenicity islands, Reptile, Salmonellosis, Virulence, Wildlife

## Abstract

The aim of this study was to estimate virulence potential of *Salmonella enterica* strains colonizing the gut of free-living sand lizards (*Lacerta agilis* L.). The strains belonged to three *Salmonella* serovars: Abony, Schleissheim, and Telhashomer. Adhesion and invasion abilities of the strains were determined in quantitative assays using the gentamicin protection method. Induction of apoptosis was assessed using HeLa cell monolayers. PCR assays were used for detection of 26 virulence genes localised within mobile elements: pathogenicity islands, virulence plasmids, and prophage sequences. In vitro studies revealed that all strains had adhesion and invasion abilities to human epithelial cells. The isolates were cytotoxic and induced apoptosis of the cells. The serovars differed in the number of virulence-associated genes: up to 18 genes were present in *Salmonella* Schleissheim, 17 in *Salmonella* Abony, whereas as few as six genes were found in *Salmonella* Telhashomer. Generally, *Salmonella* Abony and *Salmonella* Schleissheim did not differ much in gene content connected with the presence SPI-1 to -5. All of the strains lacked genes localised within bacteriophages and plasmids. The presence of virulence-associated genes and in vitro pathogenicity assays suggest that *Salmonella* sp. strains originating from autochthonous, free-living lizards can potentially infect and cause disease in humans.

**Electronic supplementary material:**

The online version of this article (10.1007/s10482-018-1079-8) contains supplementary material, which is available to authorized users.

## Introduction

The natural habitat of *Salmonella enterica* is the intestine of warm-blooded and many cold-blooded vertebrates. *S. enterica* is divided into six subspecies: *enterica*, *salamae*, *arizonae*, *diarizonae*, *houtenae*, and *indica*. Strains belonging to *S. enterica* subsp. *enterica* cause approximately 99% of *Salmonella* sp. infections in humans and warm-blooded animals, resulting in manifestations ranging from asymptomatic carriage to systemic disease (Hoelzer et al. 2011; Gal-Mor et al. 2014). Invasive, extraintestinal disease can lead to bacteraemia and systemic infections, especially in immunocompromised patients. *S*. *enterica* subsp. *enterica* comprises as many as 1586 serovars (Issenhuth-Jeanjean et al. 2014) including a few host-adapted to humans and some primates (i.e. *S*. Typhi and *S*. Paratyphi) or specific mammals or avian species (i.e. pig-associated *S.* Choleraesuis and fowl pathogen *S.* Gallinarum). However, a vast majority of others, such as *S*. Typhimurium and *S*. Enteritidis, tend to cause gastroenteritis in many different host species (Uzzau et al. 2000; Boyle et al. 2007).

The mechanism of *S. enterica* serovars pathogenicity is still unclear. Most virulence genes associated with bacterial adhesion, invasion, intravacuolar survival and extraintestinal spread are located within *Salmonella* pathogenicity islands (SPIs), plasmids and phages. To date, there have been 28 *Salmonella* pathogenicity islands detected (Yoon et al. 2015), of which 21 are characterised (Uzzau et al. 2000; Sabbagh et al. 2010). Experiments with animal models using host-specific *Salmonella* sp. have revealed that SPIs play a major role in host range and pathology of infection (Marcus et al. 2000). It has been suggested that a combination of virulence factors specific to each serovar, encoded by SPIs and virulence plasmids, is involved in the severity of salmonellosis (Andino and Hanning 2015).

Reptiles represent an important reservoir of salmonellae in nature (Geue and Löschner 2002; Briones et al. 2004) and have potential implications for public health. Although homeothermic animals and humans can contract salmonellosis from reptiles, most *Salmonella* serovars encountered in those animals have been rarely isolated from mammals and birds (Bäumler et al. 1996; Pasmans et al. 2005; Hoelzer et al. 2011).

The aim of this study was to estimate adhesion and invasion abilities to human epithelial cells, as well as cytotoxic and apoptotic activities, of *Salmonella* strains originating from sand lizards (*Lacerta agilis* L.), and to determine the presence of virulence-associated genes in their genomes.

## Materials and methods

### Bacterial strains

Eight genetically unrelated *Salmonella* strains cultured from faecal samples of free-living sand lizards *Lacerta agilis* L. (Dudek et al. 2016) were used in the study (Table 1). They were isolated in Rappaport–Vassiliadis medium and Brilliant Green Agar, and identified as *S. enterica* subsp. *enterica* serovar Schleissheim (n = 5), *S.* Abony (n = 2) and *S*. Telhashomer (n = 1), according to EN ISO 6579:2002/A1:2007 and the presence of the *invA* gene (Zając et al. 2016).

**Table 1:** Virulence-associated genes of *S. enterica* strains isolated from wild lizards

Gene	Location	*Salmonella* serovar and strain ID							
		Schleissheim					Abony		T.^a^
		J1	J6	J7	J33	J36	J23	J27	J10
*avrA*	SPI-1		♦^b^	♦		♦	♦	♦	
*invA*		♦	♦	♦	♦	♦	♦	♦	♦
*orgA*		♦	♦	♦	♦	♦	♦	♦	
*prgH*		♦	♦	♦	♦	♦	♦	♦	
*sipB*		♦	♦	♦	♦	♦	♦	♦	
*spaN*		♦	♦	♦	♦	♦	♦	♦	
*ssaQ*	SPI-2	♦	♦	♦	♦	♦	♦	♦	♦
*spiA*		♦	♦	♦	♦	♦	♦	♦	
*mgtC*	SPI-3	♦	♦	♦	♦	♦	♦	♦	♦
*siiD*	SPI-4	♦	♦	♦	♦	♦	♦	♦	♦
*sopB*	SPI-5	♦	♦	♦	♦	♦	♦	♦	♦
*sopE*	SPI 7/MPI	♦	♦	♦	♦	♦	♦	♦	
*msgA*	SPI-11	♦	♦	♦	♦	♦	♦	♦	♦
*pagC*	SPI-11	♦	♦	♦	♦	♦	♦	♦	
*cdtB*	cdtB islet/SPI11								
*lpfC*	Pathogenicity islet								
*sifA*	Pathogenicity islet	♦	♦	♦	♦	♦	♦	♦	
*sodC1*	*Gifsy2*								
*gipA*	*Gifsy1*	♦	♦	♦	♦	♦			
*bcfC*	33 kb island								
*spvC*	Virulence plasmid Virulence plasmid								
*pefA*									
*tolC*	Chromosome	♦	♦	♦	♦	♦	♦	♦	
*fyuA*	HPI								
*iutA*	Plasmid IncFIB								
*iroN*	Chromosome	♦	♦	♦	♦	♦	♦	♦	♦

^a^*T.* Telhashomer

^b^Indicates presence of a gene

### Cultivation and infection of human epithelial cells

Human epithelial cells originated from cervical cancer HeLa cell line. They were cultivated in growth medium (GM) with RPMI (Gibco), supplemented with heat-inactivated 5% fetal calf serum (FCS, Gibco), streptomycin (100 µg ml^−1^), penicillin (100 U ml^−1^) and 2 mM l-glutamine, Gibco). The cells (1 × 10^5^ ml^−1^) were seeded into 96-well plates (Greiner Bio-One) and incubation was carried out at 37 °C in humidified atmosphere with 5% CO_2_ (Nawrot et al. 2010).

Monolayers of HeLa cells were infected with *Salmonella* spp. isolates at multiplicity of infection (MOI) of 1:100 for 90 min (Suez et al. 2013). The cells were washed three times with phosphate buffered saline (PBS, Biomed) for assessment of bacterial adhesion, invasion to the cells and induction of apoptosis.

### Bacterial adhesion and invasion

Adhesion and invasion abilities were determined in quantitative assays using gentamicin protection method (Krzymińska et al. 2010). Adherence was expressed as adhesion index (InA), which designates the number of associated bacteria per 1 × 10^5^ cells. *S. enterica* invasion of epithelial cells was expressed as index (InI) defined as the number of internalised bacteria per 1 × 10^5^ HeLa cells. The index values are presented as means (standard deviation) from two experiments performed in triplicate. As controls, an invasive strain of *S.* Typhimurium ATCC 13311 and non-pathogenic *Escherichia coli* K12C600 were included.

### Cytotoxic activity of extracellular factors

Activity of cytotoxic virulence factors was analysed in bacterial filtrates. Overnight bacterial cultures in Tryptic Soy Broth (TSB, Difco) were incubated in the medium at 37 °C for 18 h with agitation at 300 rpm (Krzymińska et al. 2010; Cooley et al. 2014). The supernatants were centrifuged at 3000×*g* for 20 min and sterilised through 0.22 μm pore size membrane filters Millex-GV (Millipore). Confluent monolayers of HeLa cells were incubated with culture filtrates of *Salmonella* spp. and non-pathogenic *E. coli* K-12 C600 for 24 h at 37 °C. Cytotoxic activity to human epithelial cells was observed under an inverted microscope.

### Assessment of apoptosis

Monolayers were detached using 0.25% trypsin and 0.25% EDTA in PBS. Cell suspensions were stained with Acridine Orange (100 µg ml^−1^) and Ethidium Bromide (100 µg ml^−1^) solution, and examined under the fluorescence microscope (Nikon Eclipse TE-2000). The percentage of apoptotic cells were expressed as apoptotic index (ApI) and presented as means (standard deviation) from two experiments performed in triplicate. In positive controls, the HeLa cell monolayers were UV-B-irradiated (180 J m^−2^), whereas the cells incubated in GM comprised negative control (Ribble et al. 2005).

### Siderophore production

Cross-feeding assays with indicator strains *S.* Typhimurium TA 2700 (enterobactin and other catecholate siderophores indicator), *E. coli* LG 1522 (aerobactin and rhodotorulic acid indicator) and *Yersinia enterocolitica* 5030 (yersiniabactin indicator) were used for determination of siderophore production (Reissbrodt and Rabsch 1988; Haag et al. 1993).

### Identification of virulence genes

Virulence genes characteristic for *Salmonella* spp. were detected by PCR assays. The targeted genes encode products associated with cellular invasion (*avrA*, *invA, orgA, prgH, sipB, spaN, sopB, sopE1, gipA, cdtB, tolC*), survival within a cell (*ssaQ, sifA, pagC, spvC, spiA, mgtC, sodC1, msgA*), and adhesin or pili production (*siiD*, *lpfC*, *pefA, bcfC*). The remaining genes are associated with iron acquisition (*iroN, iutA* and *fyuA*) (Supplementary Table 1). The PCR conditions and primer sequences have been published elsewhere (Schubert et al. 1998; Skyberg et al. 2006; Huehn et al. 2010).

## Results and discussion

In this study, we characterised virulence potential of eight *Salmonella* strains isolated from autochthonous sand lizards living in natural environments in an urbanised area. The first step of bacterial colonization of host epithelium and establishment of infection is adhesion of the pathogen to the cells (López et al. 2012). All strains demonstrated the ability to adhere to human epithelial cells (Table 2). The adhesion indexes of the strains ranged from 4.7 × 10^5^ for *S.* Telhashomer J10 to 7.9 × 10^8^ CFU for *S.* Schleissheim J36. The indexes were higher than those of non-pathogenic *E. coli* K12C600 (0.12 × 10^3^), and positive control *S.* Typhimurium ATCC 13311 (7.8 × 10^4^ CFU). Several bacterial factors are involved in interactions with host receptors. López et al. (2012) have suggested that *S*. Typhimurium produce at least 13 fimbrial and three nonfimbrial adhesins. In a recent study, *S. enterica* isolates could produce adhesins including lipopolysaccharide (LPS) and SiiD protein that is recognised by Toll-like receptors.

**Table 2:** Adhesion, invasion and apoptosis indexes of *S. enterica* strains isolated from wild lizards

Index	*Salmonella* serovar and strain ID							
	Schleissheim					Abony		T*
	J1	J6	J7	J33	J36	J23	J27	J10
Adhesion index (× 10^6^)	1.03 (0.56)^a^	5.67 (2.41)	0.69 (0.27)	48.5 (10.80)	795.0 (257.30)	44.81 (21.17)	2.53 (1.28)	0.47 (0.17)
Invasion index (× 10^6^)	0.01 (0.00)^b^	3.21 (1.22)	0.18 (0.06)	0.38 (0.17)	17.82 (6.82)	23.74 (14.37)	1.97 (0.69)	0.12 (0.04)
Apoptosis index (%)	38.7 (9.6)^c^	32.3 (11.4)	15.6 (4.1)	31.2 (18.2)	63.4 (21.6)	79.5 (12.7)	29.1 (7.2)	4.1 (2.7)

*Telhashomer

^a^The number of associated (CFU) bacteria/1 × 10^5^ HeLa cells

^b^The number of internalized bacteria/1 × 10^5^ HeLa cells

^c^The percentage of apoptotic cells. All index values are presented as means (standard deviation) from two experiments performed in triplicate

All *Salmonella* tested were invasive to HeLa cells. Invasion indices ranged from 1 × 10^4^ (*S.* Schleissheim J1) to 23.7 × 10^6^ (*S.* Abony J23) and 17.8 × 10^6^ (*S.* Schleissheim J36) (Table 2). The index of *S.* Typhimurium ATCC 13311 was 8.7 × 10^5^. Non-pathogenic *E. coli* K12C600 was not invasive to HeLa cells. In epithelial cells *Salmonella* spp. strains are enclosed within vacuoles. To establish invasion to host cells, the bacteria use products of at least 29 genes located on SPI-1 (López et al. 2012). On the basis of the presence of selected virulence genes, we observed that *S. enterica* could probably invade nonphagocytic human cells by a “trigger” mechanism. The strains likely use T3SS-1 to inject the products of *sipA*, *invA*, *sopB*, and s*iiD* genes into epithelial cells. Those genes were observed in both *S.* Schleissheim and *S.* Abony. SipA effector binds directly to actin, whereas SopB activates RhoGTPases, which trigger cellular proteins that cause depolymerisation of actin. The rearrangement of the host cytoskeleton drives bacterial entry (Velge et al. 2012). SipA, SopB and InvA effector proteins could also activate signal transduction cascades, leading to chemotaxis of leucocytes and synthesis of pro-inflammatory cytokines (López et al. 2012).

All tested *Salmonella* cell-free supernatants displayed cytotoxic activity to human epithelial cells, seen as destruction of infected HeLa cells. The results suggest that the strains produced extracellular cytotoxic factors. Wang et al. (2013) have reported *Salmonella* strains producing AB5 toxins which cause signalling responses that result in secretion of proinflammatory cytokines and chemokines produced by human macrophages, epithelial and endothelial cells. The toxins consist of catalytic A- and pentameric B- subunits (Beddoe et al. 2010). Rytkönen et al. (2007) suggested that cytotoxic activity of *Salmonella* depends on the SseL effector, translocation of which is related to the SPI-2 type III secretion system. The protein SseL is similar to cysteine proteases with deubiquitinating activity.

All *Salmonella* were able to induce human epithelial cell death (Table 2). After Ethidium Bromide/Acridine Orange staining, live cells appeared green, whereas late-stage apoptotic cells are shown with orange fragmented nuclei (Supplementary Fig. 1). The highest apoptotic index was noted in cells infected with *S.* Abony J23 (79.5%) and *S.* Schleissheim J36 (63.4%), whilst the lowest was in the case of *S.* Telhashomer (4.1%). The mechanism of apoptosis involves SPI-1 effectors (López et al. 2012). Most of the analysed strains harboured SPI-1-located *sipB* encoding an effector protein that causes activation of caspase-1.

Pathogenicity of *Salmonella* is associated with the presence of virulence-related genes encoding proteins involved in colonization and survival within hosts (Huehn et al. 2010). The selected 26 virulence genes are located within mobile elements: pathogenicity islands, virulence plasmids, and prophage sequences (Table 1). The strains differed in the number of virulence-associated genes: up to 18 and 17 were present in *S*. Schleissheim and *S*. Abony, respectively, whereas only seven genes were noted in *S*. Telhashomer. Those seven genes (*invA*, *ssaQ*, *mgtC*, *siiD*, *sopB*, *msgA*, *iroN*) were present in all tested strains (Table 1).

Genes localised in SPI-1 and SPI-2, namely *sipB*, *invA*, *prgH*, *spaN*, *orgA*, *ssaQ*, and *spiA* was present in all *S*. Schleissheim and Abony strains; whereas *avrA* in all but two strains of *S*. Schleissheim. None of the SPI-1 genes was found in *S*. Telhashomer, which may indicate the absence of the island resulting in the lowest adhesion and apoptotic indexes. SPI-1 and SPI-2 genes are necessary for colonization and invasion to epithelial cells. The lack of *avrA* in two *S*. Schleissheim may be a result of recombination which often takes place in that locus (Borges et al. 2013). Moreover, in *S*. Typhi and *S*. Paratyphi, the lack of *avrA* is coincident with the ability of these strains to avoid immunological responses in the intestine, which leads to systemic infection (Prager et al. 2000). Besides, in *S*. *enterica*, even if present, *avrA* often did not express AvrA protein (Streckel et al. 2004). The *ssaQ* and *spiA* genes coding for proteins of the SPI-2 type III secretion system are essential for virulence in host cells, survival within macrophages, and biofilm formation (Dong et al. 2011).

The *mgtC* gene located within SPI-3, *siiD* within SPI-4 and *sopB* in SPI-5 were present in strains of all the three serovars. The *mgtBC* operon is necessary for inducing systemic infection in mice, SiiD secretion protein is associated with T1SS and *sopB*-encoded inositol phosphatase is involved in triggering fluid secretion secreted via SPI-1 encoded T3SS (Morgan et al. 2007).

Animal models using host-specific *Salmonella* sp. have shown that SPIs play crucial roles in host range and pathology of infection. SPI-1 and SPI-5 encode proteins appearing to have their virulence function restricted to the gut, whereas those of SPI-2, SPI-3, SPI-4 and the virulence plasmid seem to have adapted *Salmonella* spp. for growth in macrophages (Marcus et al. 2000). However, it has been reported that SPI-1-encoded SipB, SipC and SipD proteins have impact also on long-term systemic infection in mice (Lawley et al. 2006). Similarly, the AvrA efector protein is synthesized in *S*. Enteritidis not only in the intestine but also in systemic infection and may be secreted by T3SS 1 and 2 (Giacomodonato et al. 2014).

The *msgA* and *pagC* genes located within SPI-11, promoting survival within macrophages, were present in all isolates except for *pagC* absent in *S*. Telhasomer. SPI-11 has a mosaic structure and therefore its parts can be present or absent in genomes of *S.* Typhimurium and *S*. Typhi strains (Morgan 2007).

Generally, we noticed that strains of serovars Abony and Schleissheim did not differ in gene content connected with SPI-1 to -5 and *pagC* (SPI-11) presence. Suez et al. (2013) analysed virulence gene profiles of invasive non-typhoidal *Salmonella* and allocated genes of SPI-1-5, -9, -13, -14 to the core part of the genomes.

The *tolC*, *sifA*, and *gipA* genes were present in all *S*. Schleissheim strains and *sifA* and *tolC* in *S.* Abony. The *sifA* gene has been encountered among genes usually absent from invasive non-typhoidal *Salmonella* serovars and required probably for adaptation of some serovars to a specific homoeothermic hosts (Suez et al. 2013). Apart from siderophore receptor genes *iutA* and *fyuA,* five genes were absent in all strains examined: *sopE*, *lpfC*, *sodC1*, *bcf*, *spvC*, and *pefA*. Absence of a virulence gene may suggest that it is not essential for invasive manifestation in humans as was suggested for the lack of *sopE* located within SopEφ on SPI-7, encoding effector protein for SPI-1 T3SS, in the case of 80% invasive non-typhoidal strains originated from human blood (Suez et al. 2013). On the other hand the gene was noted in all *S*. Enteritidis strains isolated from broiler meat and slaughterhouse (Borges et al. 2013), whereas *sopE* was demonstrated only in 24% of genomes of *Salmonella* isolated from captive lizards (Pasmans et al. 2005). Plasmid-mediated *pefA* and *spvC* genes were absent from isolates of all three serovars, suggesting that they can be encoded by the same virulence plasmid (Skyberg et al. 2006). In mammals and birds, the *spv* virulence locus is required for sustained extra-intestinal infections and clinical disease through macrophage cytotoxicity, and destabilisation of the cytoskeleton of the eukaryotic cells. In strains isolated from captive lizards, it has been present in a single *Salmonella* strain, which coincided with the limited number of extra-intestinal infections in lizards and seems not to be crucial for sustained colonization (Pasmans et al. 2005).

In the genomes of *S*. Schleissheim and *S*. Abony, we found typhoid-associated virulence gene *cdtB*. The presence of *cdtB* has been primarily related to human *Salmonella* isolates (Haghjoo and Galan 2004). However, Skyberg et al. (2006) have reported the gene in *Salmonella* mainly associated with avian salmonellosis and isolated from healthy birds.

All *Salmonella* serovars produced a catecholate siderophore and had a receptor gene (*iroN*) for salmochelin, which is glycosylated enterobactin. Salmochelin is not susceptible to lipocalin-2, a protein preventing bacterial iron acquisition. Lipocalin-2 resistance mediated by *iroN* confers a specific benefit during growth of *S.* Typhimurium in inflamed cecum of mice (Raffatellu et al. 2009).

*S*. Abony, Schleissheim and Telhashomer are not often encountered in mammals and birds (Bäumler et al. 1996; Uzzau et al. 2000). *S.* Abony was detected with 6.2% frequency in Mediterranean turtle *Testudo graeca* faeces (Briones et al. 2004) and it was occasionally associated with human salmonellosis (Hall and Rowe 1992; Woodward et al. 1997). *S.* Schleissheim was identified in cattle (Wieczorek and Osek 2013) and mistle thrush *Turdus viscivorus* (Hernandez et al. 2003). Human salmonellosis caused by this serovar has been previously reported in Turkey (Aksoycan et al. 1983) and Poland (PZH and GIS 2016). Both serovars occur occasionally in Poland in wildlife and food-producing animals, as well as organic fertilizers (Skarżyńska et al. 2017). *S.* Telhashomer has been identified more frequently than other serovars in toads (Sharma et al. 1977), but no reports of human cases was noted. In human-influenced environments, *Salmonella* spp. found in wild animals might coincide with serovars disseminated to the habitat (Palmgren et al. 2000).

The results of this study showed that free-living sand lizards occurring in common urban locations can be carriers of pathogenic *Salmonella*, as the strains revealed adhesion, invasion, cytotoxicity and induced apoptosis of human epithelial cells, although the serovars Schleissheim, Abony and Telhashomer differed in their virulence gene profiles. They may pose a disease threat if the bacterium is transferred from clinically healthy native reptiles into birds, mammals and humans.

## Electronic supplementary material

Below is the link to the electronic supplementary material.

Supplementary material 1 (PDF 332 kb)
