# Supplementary material for: In vitro virulence characteristics of rare serovars of Salmonella enterica isolated from sand lizards (Lacerta agilis L.)
Source: Antonie Van Leeuwenhoek. 2018 May 19;111(10):1863–70. doi: 10.1007/s10482-018-1079-8 (PMC6153992; doi:10.1007/s10482-018-1079-8)
Supplement: Supplementary file 1 — Supplementary material 1 (PDF 332 kb) [file 10482_2018_1079_MOESM1_ESM.pdf]

SUPPLEMENTARY MATERIAL

In vitro virulence characteristics of rare serovars of *Salmonella enterica* isolated from sand lizard (*Lacerta agilis* L.)

Joanna Mokracka[1]*, Sylwia Krzymińska[1], Danił Ałtunin[1], Dariusz Wasyl[2], Ryszard Koczura[1], Krzysztof Dudek[3], Monika Dudek[4], Zofia Anna Chyleńska[5], and Anna Ekner-Grzyb[6]

[1] Department of Microbiology, Faculty of Biology, Adam Mickiewicz University in Poznań, Umultowska 89, 61-614 Poznań, Poland,

[2] Department of Microbiology, National Veterinary Research Institute, Partyzantów 57, 24-100 Puławy, Poland

[3] Department of Zoology, Institute of Zoology, Poznań University of Life Sciences, Wojska Polskiego 71 C, 60-625 Poznań, Poland,

[4] Laboratory of Neurobiology, Institute of Zoology, Poznań University of Life Sciences, Wojska Polskiego 71 C, 60-625 Poznań, Poland,

[5] Department of Nature Education and Conservation, Faculty of Biology, Adam Mickiewicz University in Poznań, Umultowska 89, 61-614 Poznań, Poland,

[6] Department of Plant Ecophysiology, Faculty of Biology, Adam Mickiewicz University in Poznań, Umultowska 89, 61-614 Poznań, Poland,

* **Corresponding author**: Joanna Mokracka, E-mail address: amok@amu.edu.pl; phone: +48 61 8295939

Supplementary Table 1 Function and location of *Salmonella enterica* virulence genes investigated

Gene	Function	Location	Reference
avrA	cysteine protease(inhibits proinflamatory anti-apoptotic NF-kappa B pathway)	SPI-1	Streckel et al. 2004
invA	invasion protein, host recognition/invasion		Lara-Tejero and Galán 2009
orgA	putative component of T3SS, host recognition/invasion		Klein et al. 2000
prgH	host recognition/invasion		Klein *et al*. 2000
sipB	entry into nonphagocytic cells, killing of macrophages		Lara-Tejero and Galán 2009
spaN	entry into nonphagocytic cells, killing of macrophages		Lara-Tejero and Galán 2009
ssaQ	T3SS protein	SPI-2	Kuhle and Hensel 2004
spiA	outer-membrane component of the SPI-2 T3SS, survival within macrophage, biofilm formation		Dong et al. 2011
mgtC	intramacrophage survival protein	SPI-3	Blanc-Potard et al. 1999
siiD	HLYD family secretion protein	SPI-4	Morgan et al. 2007
sopB	effector protein secreted via T3SS-1 host recognition/invasion	SPI-5	Wood et al. 1998
sopE	T3SS effector protein	SPI-7/MPI	Ehrbar and Hardt 2005

**Gene**	**Function**	**Location**	**Reference**
msgA	survival within macrophages	SPI-11	Morgan 2007
pagC	survival within macrophages	SPI-11	Gunn et al. 1995
cdtB	host recognition/invasion	cdtB islet/SPI11	Haghjoo and Galan 2004
lpfC	host recognition/invasion	pathogenicity islet	Weening et al. 2005
sifA	Efector protein of T3SS-2 Filamentous structure formation	pathogenicity islet	Kuhle and Hensel 2004
sodC1	periplasmic Cu, Zn-superoxide dismutase	*Gifsy2*	Fang et al. 1999
gipA	Peyer's patch-specific virulence factor	*Gifsy1*	Stanley et al. 2000
bcfC	bovine colonization factor	33-kb island	Weening et al. 2005
spvC	growth and survival within the host	virulence plasmid	Sabbagh et al. 2010
pefA	host recognition/invasion		Bäumler et al. 1996
tolC	host recognition/invasion	chromosome	Skyberg et al. 2006
fyuA	yersiniabactin receptor protein	HPI	Schubert et al. 1998
iutA	aerobactin receptor protein	IncFIB plasmid	Han et al. 2012
iroN	salmochelin receptor protein	chromosome	Sorsa et al. 2003

REFERENCES

Blanc-Potard AB, Solomon F, Kayser J, Groisman EA (1999) The SPI-3 pathogenicity island of *Salmonella enterica*. J Bacteriol 181:998–1004

Ehrbar K, Hardt W (2005) Bacteriophage-encoded type III effectors in *Salmonella enterica* subspecies 1 serovar Typhimurium. Infect Genet Evol 5:1–9

Fang FC, DeGroote MA, Foster JW, Bäumler AJ, Ochsner U, Testerman T, Bearson S, Giárd JC, Xu Y, Campbell G, Laessig T (1999) Virulent *Salmonella typhimurium* has two periplasmic Cu, Zn-superoxide dismutases. Proc Natl Acad Sci USA 96:7502–7507

Gunn J, Alpuche-Aranda C, Loomis W, Belden W, Miller S (1995) Characterization of the *Salmonella typhimurium pagC/pagD* chromosomal region. J Bacteriol 177:5040–5047

Han J, Lynne AM, David DE, Tang H, Xu J, Nayak R, Kaldhone P, Logue CM, Foley SL (2012) DNA sequence analysis of plasmids from multidrug resistant *Salmonella enterica* serotype Heidelberg isolates. PLoS ONE **7**: e51160

Klein JR, Fahlen TF, Jones DB (2000) Transcriptional organization and function of invasion genes within *Salmonella enterica* serovar Typhimurium pathogenicity island 1, including the *prgH*, *prgI*, *prgJ*, *prgK*, *orgA*, *orgB*, and *orgC* genes. Infect Immun 68:3368–3376

Kuhle V, Hensel M (2004) Cellular microbiology of intracellular *Salmonella enterica*: functions of the type III secretion system encoded by *Salmonella* pathogenicity island 2. Cell Mol Life Sci 61:2812–2826

Lara-Tejero M, Galán JE (2009) *Salmonella enterica* serovar Typhimurium pathogenicity island 1-encoded type III secretion system translocases mediate intimate attachment to nonphagocytic cells. Infect Immun 77:2635–2642

Sorsa LJ, Dufke S, Heesemann J, Schubert S (2003) Characterization of an *iroBCDEN* gene cluster on a transmissible plasmid of uropathogenic *Escherichia coli*: evidence for horizontal transfer of a chromosomal virulence factor. *Infect Immun* 71:3285–3293

Stanley TL, Ellermeier CD, Slauch JM (2000) Tissue-specific gene expression identifies a gene in the lysogenic phage Gifsy-1 that affects *Salmonella enterica* serovar Typhimurium survival in Peyer’s patches. J Bacteriol 182:4406–4413

Weening EH, Barker JD, Laarakker MC, Humphries AD, Tsolis RM, Bäumler AJ (2005) The *Salmonella enterica* serotype Typhimurium *lpf, bcf, stb, stc, std,* and *sth* fimbrial operons are required for intestinal persistence in mice. Infect Immun 73:3358–3366

Wood MW, Jones MA, Watson PR, Hedges S, Wallis TS, Galyov EE (1998) Identification of a pathogenicity island required for *Salmonella* enteropathogenicity. Mol Microbiol 29:883–891

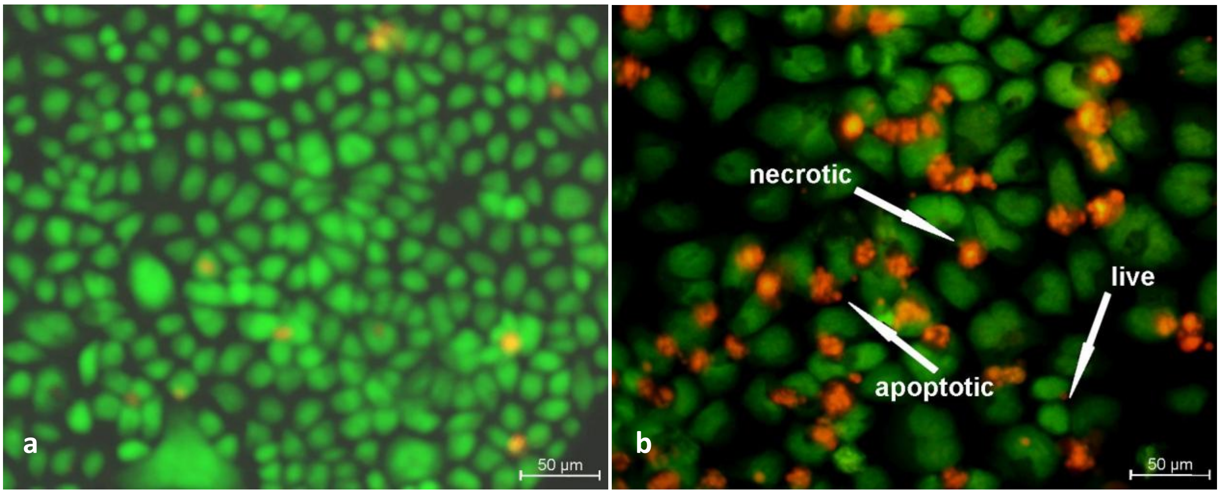

Supplementary Fig 1 HeLa cells stained with AO/EB and observed by fluorescence microscopy: **a**) Non-infected HeLa cells **b)** HeLa cells infected with *S.* Schleissheim J6. Arrows indicate live (green), apoptotic (red with fragmented nuclei), and necrotic (red) cells
